# MRI T_2_ relaxometry of brain regions and cognitive dysfunction following electroconvulsive therapy

**DOI:** 10.4103/0019-5545.37321

**Published:** 2007

**Authors:** Girish Kunigiri, P. N. Jayakumar, N. Janakiramaiah, B. N. Gangadhar

**Affiliations:** Bradgate Mental Health Unit, Leicester, UK; * Department of Neuroradiology and Imaging, National Institute of Mental Health and Neurosciences, Bangalore, Karnataka, India; ** Department of Psychiatry, National Institute of Mental Health and Neurosciences, Bangalore, Karnataka, India

**Keywords:** Depression, electroconvulsive therapy, memory orientation, MRI

## Abstract

**Background:**

Although electroconvulsive therapy (ECT) causes no structural brain damage, recent studies reported altered brain perfusion acutely following ECT. This is in keeping with brain edema which was noted in animal experiments following electroconvulsive shock.

**Aim:**

This study examined alteration in magnetic resonance imaging (MRI) T_2_ relaxation time, a measure of brain edema, and its relation to therapeutic efficacy, orientation and memory impairment with ECT.

**Materials and Methods:**

Fifteen drug-naive consenting patients of major depressive disorder with melancholia (DSM-IV) received ECT as first-line treatment. MRI scans were done before the first ECT and at 2 hours after the second ECT. T_2_ relaxation time was measured bilaterally in thalamus, hippocampus, medial temporal lobes and dorsolateral frontal cortex by a blind rater.

**Results:**

Depression scores and memory scores were reduced significantly both after the second and fifth ECT. There was no change in T_2_ relaxation time after second ECT.

**Conclusion:**

The finding suggests that ECT does not produce demonstrable change acutely in brain parenchyma detectable by MRI scans.

Electroconvulsive therapy (ECT) produces therapeutic response in major psychiatric illnesses, as well as adverse effects. Until recently, neuroimaging studies failed to show any long-term effects of ECT on brain structure.[1–5] Mandler *et al.*[6] and Scott *et al.*[7] showed an increase in magnetic resonance imaging (MRI) T_1_ relaxation time (a measure of brain water content) acutely after ECT. In a pilot study by Diehl *et al.*,[8] MRI T_2_ relaxation time increased within 2 hours after the second ECT and correlated with short-term memory impairment, though at trend level. However, their sample size was small (*n* = 5) and they had examined only the effects of unilateral electrode placement. Their study did not examine hippocampus and dorsolateral frontal cortex, which are implicated in memory. We hypothesized that ECT produced increased brain edema (detectable by MRI T_2_ relaxation time) acutely, and this increase correlated with disorientation and memory impairment following ECT.

## MATERIALS AND METHODS

### Subjects

Fifteen (seven males) consecutive and consenting right-handed patients with major depressive episode with melancholic features[9] formed the sample. The severity of depression was rated twice weekly using the Hamilton Rating Scale for Depression (HRSD).[10] The mean (SD) HRSD at baseline was 26.6 (4.5). Thirteen (85%) were in their first episode. The mean (SD) duration of the current episode was 19 (15.6) weeks. The mean (SD) age of the sample was 31.6 (6.5) years (range = 24-46 years). None had ever received medications or ECT. All had seven years or more of formal education and scored 28 on mini-mental state examination.[11] Patients with mental retardation, medical/neurological disease known to affect cognitive functions or who qualified for alcohol and drug abuse/ dependence were excluded from the study.

### Orientation and memory tests

The same clinician (GK) without the knowledge of ECT laterality conducted all assessments. Orientation was assessed using Orientation Battery Test (OBT)[12] and trail-making test (TMT Form-A).[13] Orientation was assessed at baseline (within 48 hours before first ECT) and after 20 minutes, 50 minutes, 2 hours and 8 hours following the second and fifth ECT sessions. Retrograde memory was assessed by verbal paired associates using the Wechsler Memory Scale.[14] At least six words out of ten pairs had to be recalled 24 hours after the learning session for inclusion in the study. Anterograde verbal memory was tested using verbal learning test and passage test.[13–15] In verbal learning test, a minimum of 8 out of 12 words had to be recalled after 15 minutes for inclusion in the study. Anterograde nonverbal memory was tested using Benton Visual Retention Test (BVRT).[16] Parallel forms were used at different occasions. Memory tests were performed within 48 hours of the first ECT and at 8 hours after the second and fifth ECT.

### ECT procedure

ECT was administered three times a week under general anesthesia using thiopentone (3 mg/kg), succinylcholine (0.75 mg/kg) and atropine (0.65 mg). No patient received psychotropic medications during the course of ECT, except for two patients who required lorazepam 2 mg at bedtime. The treating psychiatrist chose ECT stimulus laterality. Ten patients received bilateral (BL) while five received right unilateral (UL) ECT. Threshold (T) was assessed at the first ECT session using titration method. The stimulus dose at subsequent sessions was modestly suprathreshold (T + 60 mC) in BLECT and moderately suprathreshold (2.5 × T) in ULECT. All had adequate seizures with single stimulus at second ECT session. Motor (cuff method)[17] and EEG (F_3_ and F_4_ channels referenced to ipsilateral mastoids) seizure durations were recorded at all ECT sessions. Four patients (two each from UL and BLECT groups) at the second ECT and none at the fifth ECT had prolonged seizures (EEG ≥ 120 seconds). Prolonged seizure was terminated by 5-10 mg intravenous diazepam. Two patients each had emergent delirium after the second ECT (both BLECT patients) and the fifth ECT (both ULECT patients). It was managed by thiopentone (50-75 mg) administered intravenously.

### MRI studies

MRI of the brain was done within 48 hours preceding the first ECT and at 2 hours after the second ECT. 1.5-tesla superconducting system was used in all patients. A sagittal scout series (T_1_ -weighted, 5-mm slice thickness and 2.0 mm interslice space) was performed to confirm consistent positioning. All patients underwent routine MRI evaluation including T_1_-weighted sagittal, proton density and T_2_ coronal and Inversion Recovery protocols. Data for T_2_ quantification was collected with dual echo-multiplanar (DEMP) sequences.

Regional T_2_ values were determined using proprietary software. For each echo-time of both DEMP sequences, the signal intensity was recorded for a 20-mm^3^ uniformly defined circular region of interest (ROI) placed within each selected brain region. A regional T_2_ value for each DEMP sequence was then estimated by proprietary software inbuilt in the equipment. No effort was made to correct the values obtained with reference to background air or cranial vault. Mean T_2_ values were used for analysis. MRI T_2_ relaxation time was measured from each scan by an experienced neuroradiologist (JPN) who was unaware whether the particular scan was pre- or post-ECT scan and whether the patient received UL or BLECT.

The MRI T_2_ relaxation times of five regions of interest (ROIs) were measured in both the cerebral hemispheres. The ROI included thalamus, hippocampus (HC), medial temporal lobe gray matter (MTLGM), medial temporal lobe white matter (MTLWM) and dorsolateral frontal cortex (DLFC) as they are implicated in memory.[13]

**Figure 1:**
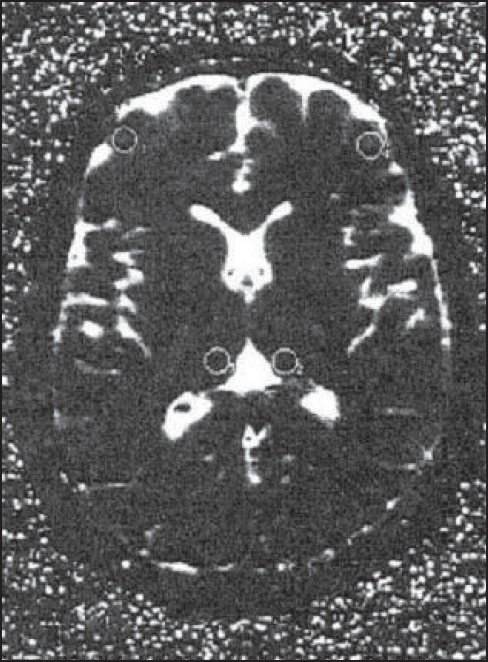
T_2_ weighted image showing thalamus (ROI 1& 2) and dorsolateral frontal cortex (ROI 3 & 4)

**Figure 2:**
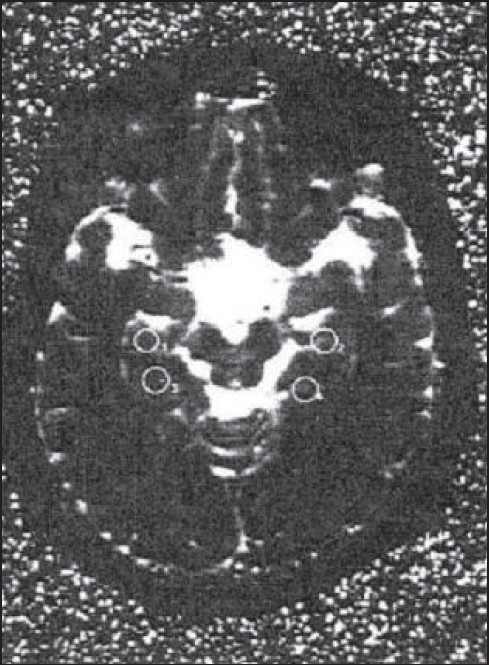
T_2_ weighted image showing medial temporal lobe gray matter (ROI 1& 2) and hippocampus (ROI 3 & 4)

Thalamus was identified on the axial slice through the mid-level of the diencephalon medial to the posterior limb of internal capsule [Figure 1]. The MTLGM and HC were sampled on the axial slice through the rostral midbrain containing the substantia nigra, cerebral aqueduct and superior colliculi [Figure 2]. The MTLWM ROI was placed on the same axial slice in the white matter just lateral to the MTLGM. The DLFC measurements were taken at the gray matter of midfrontal gyrus on a transverse slice passing through the levels of frontal horns and the trigone [Figure 1].

### Statistical methods

Change in HRSD scores across the ECT course was tested using one-way RMANOVA. Changes in orientation scores across various recording points after ECT were measured using one-way RMANOVA separately at the second and fifth ECT sessions. Orientation scores at 20 minutes after the second and fifth ECT were compared using paired t-test. Impairment in memory test scores during the ECT course was tested using one-way RMANOVA.

As UL and BLECT patients did not differ with respect to MRI T_2_ relaxation time before or after ECT, UL and BLECT patients have been analyzed together. Change in regional MRI T_2_ relaxation time after ECT was tested using paired t-test. Significance (α) was set at *P* < 0.05.

## RESULTS

Mean HRSD scores significantly dropped over the two-week assessment period [Table 1]. Orientation scores dropped at 20 minutes following ECT and recovered over time in the next 2 hours at both the ECT sessions [Table 1]. Orientation score was lower at 20 minutes after the fifth ECT than the corresponding score after the second ECT in the Orientation Battery Test (*t* = 3.25, *P* < 0.01; Table 1). Memory scores decreased in all the areas measured over the course of ECT with cumulative effect [Table 2].

**Table 1:** Mean (SD) of HRSD and orientation scores (*n* = 15)

Variables	Assessment period					One-way RMANOVA
		
	0 week	0.5 week	1 week	1.5 week	2 week	*F*; df; *P*
HRSD scores	26.6 (4.5)	20.2 (5.2)	12.8 (5.1)	7.3 (5.3)	5.2 (3.9)	83.63; 4.56; <0.001
	**Pre-ECT#1**	**20 minutes**	**50 minutes**	**2 hours**	**8 hours**	
		
Second ECT						
OBT*	12 (0.0)	6.6 (2.7)	9.6 (1.6)	10.8 (1.4)	11.8 (0.4)	36.8; 4.56; <0.001
TMT (sec)**	66.8 (27.3)	139.6 (78.5)	92.2 (63.9)	63.9 (18.9)	61.9 (20.3)	9.09; 4.56; <0.001
Fifth ECT						
OBT	-	4.9 (3.0)	9.3 (2.0)	10.3 (2.6)	11.7 (0.6)	38.4; 3.42; <0.001
TMT (sec)		135.1 (109.4)	98.1 (71.6)	74.8 (43.3)	60.8 (19.8)	4.63; 3.42; <0.001

OBT = Orientation battery test; TMT = Trail-making test (Form-A),

* Higher scores indicate better orientation;

** Shorter the time taken to complete the task, better is the orientation

**Table 2:** Mean (SD) of memory scores* (*n* = 15)

Variables	Time interval		One-way RMANOVA	
		
	Pre-ECT#1	Post-ECT#2	Post-ECT#5	*F*; df; *P*
Verbal paired associate	9.1 (0.9)	5.9 (1.4)	5.3 (1.8)	32.8; 2.28; <0.001
Verbal learning test	9.8 (1.0)	6.2 (2.6)	4.3 (1.6)	30.7; 2.28; <0.001
Passage test	14.9 (1.7)	15.7 (3.3)	9.6 (3.5)	24.9; 2.28; <0.001
Benton visual retention test	8.8 (0.8)	8.3 (0.8)	7.7 (1.5)	7.2; 2.28; 0.03

* Lower scores indicate memory impairment

There was no significant change in MRI T_2_ relaxation time with ECT in any of the ROIs studied 2 hours after second ECT [Table 3]. Since there was no observed alteration in MRI T_2_ relaxation time with ECT, no attempt was made to correlate this with therapeutic efficacy, orientation and memory scores.

**Table 3:** Pre- to post-ECT mean ±; SD (range) MRI T_2_ relaxation time (msec) (*n* = 15)

ROI		Pre-ECT	Post-ECT
Thalamus	Right	105±3.8 (99-112)	106.5±5.0 (97-114)
	Left	107.0±4.0 (101-116)	108.5±4.0 (102-117)
HC	Right	149.8 ± 12.7 (131-175)	146.7 11.3 (130-165)
	Left	149.8 ± 11.7 (129-170)	147.6 ± 12.3 (123-164)
MTLGM	Right	125.7 ± 9.0 (110-142)	122.9 ± 10.2 (107-145)
	Left	124.1 ± 11.4 (100-147)	121.6 ± 10.6 (102-141)
MTLWM	Right	107.6 ± 6.3 (100-127)	109.3 ± 6.4 (94-118)
	Left	109.3 ± 8.8 (88-127)	107.0 ± 10.5 (92-123)
DLFC	Right	103.5 ± 4.7 (97-114)	103.6 ± 5.8 (95-115)
	Left	103.7 ± 4.3 (96-114)	103.9 ± 7.6 (93-124)

There was no statistically significant change in MRI T_2_ relaxation time after second ECT using paired *t*-test

## DISCUSSION

ECT produces no lasting brain damage, although a few studies have shown evidence of brain edema immediately following ECT.[6–8] Our sample was homogenous and all patients were drug naive and nearly all (85%) had first episode of depression. ECT procedures followed contemporary standards (modified, brief pulse, EEG monitored). Parallel forms of short-term memory tests were used at different occasions. This minimized the bias of practice effect, and this study ensured blind design while rating or testing memory functions. Patients improved from depression with ECT. Disorientation occurred immediately after the second and fifth ECT sessions. As in earlier studies, disorientation was more pronounced after the fifth ECT, suggesting cumulative effects.[18,19] There was significant memory impairment following ECT, as reported earlier.[19–21]

MRI scans done before ECT did not reveal any abnormality in the gray or white matter. Similarly Videbech *et al*[22] reported no structural abnormality of the brain in young depressives (*n* = 42; mean age 42 years). MRI scan was done 2 hours after the second ECT. The second ECT session was chosen as it avoids multiple stimuli as in the first ECT session. Two-hour criterion was chosen based on previous reports, which have suggested that change in T_2_ relaxation times was maximum approximately 2 hours after ECT.[6,7] However, both the earlier studies had patients who were above 50 years. Some of the limitations of these studies included consideration of total brain area for measurement of T_1_ and T_2_ relaxation time and had used a lower MRI magnetic field strength (0.08 tesla). Patients were scanned immediately after ECT (within 15 minutes), and T_2_ relaxation time changes are not expected to occur by then. In our study T_2_ relaxation time was used to measure the water content in the brain tissue following ECT, as it is more sensitive than T_1_.[23] While reading the MRI, the neuroradiologist was not aware of stimulus laterality and whether the scan was done before or after ECT. Examinations were made with 1.5 tesla MRI system, unlike in earlier studies.[6,7]

The brain regions selected (thalamus, MTLGM and MTLWM) in both the hemispheres for this study were similar to those in the study by Diehl *et al*.[8] In addition, other regions of interest such as HC and DLFC were also studied, in view of their role in memory function.[13] There was no significant change in MRI T_2_ relaxation time in any of the regions between pre- and post-ECT [Table 3]. Change in T_2_ relaxation time was also absent in the earlier study, which examined the whole brain.[7] Diehl *et al.*,[8] too, found no change in T_2_ relaxation time in four of the six regions studied. The difference in their study, which was observed in two regions, was significant only with one-tailed t-test, suggesting a possible type-I error. When unilateral ECT patients alone were analyzed, there was no significant difference in T_2_ relaxation time following second ECT.[24] Even the most sensitive techniques (3D high-resolution magnetic resonance imaging) failed to detect changes in the cerebral structure immediately after ECT.[5] It is possible that ECT did not produce any structural change in brain detectable on routine MRI examination, or increase in brain water content (edema) may not be of sufficient magnitude to be detected in MRI T_2_ relaxation time after the second ECT.

Since there was no alteration in the T_2_ relaxation time following ECT, no correlation with clinical effects was attempted. In a pilot study by Diehl *et al.*,[8] MRI T_2_ relaxation time increased within 2 hours after the second ECT and correlated with short-term memory impairment, though at trend level. However, their sample size was small (*n* = 5) and they had examined only the effects of unilateral electrode placement. It is known that BLECT produces greater memory impairment, and prolonged seizure may be more deleterious. However, T_2_ relaxation times did not change significantly in the 10 patients receiving even BLECT (mean T_2_ relaxation time of all ROIs; pre-ECT 119.3 ± 3.8 and post-ECT#2 117.7 ± 6.3; *t* = 1.04, *P* = 0.32). Nor was the change significant in the four patients who had prolonged seizures (mean T_2_ relaxation time of all ROIs; pre-ECT 118.3 ± 5.1 and post-ECT#2 117.3 ± 3.1; *t* = 0.34, *P* = 0.75). It is arguable that the change in T_2_ values would occur with more ECTs as a cumulative effect. But this seems unlikely, as earlier studies failed to indicate the same.[6,7]

The results are reassuring. ECT does not produce acute morphological changes in the brain detectable by routine MRI techniques. This probably suggests that the mechanism of memory impairment following ECT may not be detectable by the methods used in the current study for structural evaluation of the brain. Diffusion-weighted scan, a more sensitive MRI method for recognition of abnormal proton changes, may permit detection of ECT-induced brain edema.

In conclusion, ECT is effective in depression. As expected, it resulted in disorientation and memory impairment with cumulative effects. There was no demonstrable structural change in the brain identified by MRI T_2_ relaxation time after second ECT. ECT-induced structural change, if any, may not be of sufficient magnitude to be detectable by routine MRI T_2_ imaging protocols. More sensitive MR techniques may be recommended for future research.
